# Association between Cardiac Autonomic Neuropathy and Coronary Artery Lesions in Patients with Type 2 Diabetes

**DOI:** 10.1155/2020/6659166

**Published:** 2020-12-30

**Authors:** Lei Liu, Qiansheng Wu, Hong Yan, Baoxian Chen, Xilong Zheng, Qiang Zhou

**Affiliations:** ^1^Division of Cardiology, Department of Internal Medicine, Tongji Hospital, Tongji Medical College, Huazhong University of Science and Technology, Wuhan, China; ^2^Division of Cardiothoracic and Vascular Surgery, Tongji Hospital, Tongji Medical College, Huazhong University of Science and Technology, Wuhan, China; ^3^Department of Biochemistry and Molecular Biology, Cumming School of Medicine, Libin Cardiovascular Institute of Alberta, University of Calgary, Alberta, Canada

## Abstract

**Objective:**

Cardiac autonomic neuropathy (CAN) is a common and serious complication of diabetes mellitus with various systemic involvements, such as atherosclerotic cardiovascular disease. We aimed to evaluate the association between CAN and coronary artery lesions in patients with type 2 diabetes. *Research Design and Methods*. We retrospectively reviewed the medical records of 104 patients with type 2 diabetes and coronary artery disease (CAD). We evaluated heart rate variability (HRV) parameters (SDANN, SDNN, and pNN50) to assess cardiac autonomic function. The severity of coronary lesions was assessed by the Gensini scores and the number of affected vessels. Correlation analyses between HRV parameters and the severity of coronary lesions and clinical parameters were performed.

**Results:**

Spearman's correlation analysis showed a significant negative correlation between SDANN and Gensini scores (*r* = −0.22, *P* = 0.03). Interestingly, this finding remained significant after adjusting for clinical covariates (*r* = −0.23, *P* = 0.03). However, there was no association between HRV parameters and the severity of coronary lesions as assessed by the number of affected vessels. Clinical parameters were not significantly correlated with HRV parameters (all *P* > 0.05).

**Conclusions:**

Cardiac autonomic neuropathy might be related to the degree of coronary atheromatous burden in patients with type 2 diabetes. Screening for cardiac autonomic neuropathy might potentially be beneficial in the risk stratification of patients with type 2 diabetes.

## 1. Introduction

Diabetes mellitus has emerged as a major threat on patient survival and quality of life, with huge, socioeconomic, and social challenges [1]. The prevalence of diabetes mellitus is rapidly increasing due to an increased burden of obesity and related risk factors, such as increased consumption of highly processed foods, socioeconomic development, and a decrease in physical activity [2]. Type 2 diabetes accounts for 95% of diagnosed diabetes mellitus and is projected to affect hundreds of millions of people globally in the next few decades. Most patients with diabetes mellitus manifest at least one of its complications. Diabetes mellitus has both macrovascular and microvascular complications, which become more prevalent with increased disease duration [3].

An increasing body of evidence has shown that type 2 diabetes is a common chronic disease that is associated with substantial morbidity and mortality due to coronary artery disease (CAD), which is a macrovascular complication of diabetes mellitus [4]. Moreover, cardiac autonomic neuropathy (CAN) is a common and serious complication of diabetes mellitus with various systemic involvements, resulting in atherosclerotic cardiovascular disease [5]. Diabetic autonomic neuropathy is implicated in the pathogenesis of vascular damage and subsequent coronary artery disease, which may result in disabling clinical and functional manifestations. Recently, studies that dysfunction of the cardiac autonomic system may be effective in the development of vascular atherosclerosis have been well documented [6, 7]. There is a lot of evidence that cardiac autonomic neuropathy plays a crucial role in the pathogenesis of progressive vessel atherosclerosis [8, 9]. Furthermore, diabetic autonomic neuropathy is closely associated with subclinical myocardial dysfunction, interstitial myocardial fibrosis, and metabolic changes [10, 11]. Furthermore, CAN also significantly increases the incidence of cardiovascular disease and its related mortality. However, the independent relationship of cardiac autonomic neuropathy with the severity of coronary lesions in type 2 diabetic patients has not been established.

Heart rate variability (HRV), which reflects the status of the cardiac sympathovagal balance, is a noninvasive, practical, and reproducible index used to assess cardiac autonomic function [12, 13]. Recently, more attention has been placed on HRV assessment as a diagnostic tool in the prediction of prognosis in various neurological disorders and in the evaluation of autonomic impairment [14]. Numerous studies have demonstrated changes in HRV patterns are associated with the cardiac autonomic system in subjects with physiological and pathological conditions, particularly in type 2 diabetic patients [15].

There is increasing evidence that reduced HRV may be related to worse cardiovascular outcome among patients with type 2 diabetes [16, 17]. However, little is known regarding the relationship between the severities of coronary artery lesions and CAN in patients with type 2 diabetes. Therefore, we aimed to investigate the association between HRV and the severity of coronary artery lesions in patients with type 2 diabetes and CAD.

## 2. Methods

### 2.1. Study Population

We conducted a retrospective study on 104 patients with type 2 diabetes and CAD admitted to the Tongji Hospital in Wuhan (China) from July 2017 to October 2019. All data were extracted and reviewed from the patient medical records. In addition, CAN diagnosis was made based on medical records. We used the guidelines of the CAN Subcommittee of the Toronto Consensus Panel on Diabetic Neuropathy for CAN diagnosis and staging [18]. None of the participants received antiarrhythmic therapy such as beta-blockers or calcium channel blockers prior to 24-hour dynamic electrocardiogram; therefore, their HRV was not affected by the above-mentioned therapy. Patients were diagnosed with type 2 diabetes based on their medical history or according to the American Diabetes Association criteria as previously reported [19]. Detailed diagnostic criteria for CAD have been previously described [20]. Precisely, CAD was defined as the presence of at least one significant coronary artery stenosis with luminal diameter > 50%on coronary angiography. The severity of atherosclerosis was assessed using the Gensini scores and the number of diseased vessels (stenosis > 50%). The Gensini scores were calculated using a method previously described [21]. In each coronary segment, the narrowing of the coronary artery lumen was rated 1, 2, 4, 8, 16, and 32 for 0–25%, 26–50%, 51–75%, 76–90%, 91–99%, and 100% stenosis, respectively. Then, a multiplier was assigned to each segment depending on the prognostic importance of the area supplied by that segment: 5 for the left main coronary artery, 2.5 for the proximal left anterior descending coronary artery (LAD), right coronary artery, and proximal left circumflex branch, 1.5 for the midsegment of LAD, 0.5 for the second diagonal branch and posterolateral branches, and 1 for the other branches. The final Gensini score for each patient was the sum of the total scores for all affected segments.

We excluded patients with comorbidities, such as valvular heart disease, rheumatic diseases, connective tissue disease, severe liver and kidney dysfunction, hyperthyroidism, and infection. Moreover, we excluded patients with chronic obstructive coronary lesions, previous myocardial infarction, or coronary artery bypass grafting.

Ethics committee review was not necessary because this study was a retrospective analysis. Informed consent was waived because this study involved an analysis of existing medical records with neither breach of privacy nor interference with clinical decisions related to patient care. The study was conducted in accordance with the principles of the Declaration of Helsinki.

### 2.2. Clinical and Paraclinical Evaluation

Demographic factors, including sex, age, height, weight, ethnicity, cigarette smoking, alcohol consumption, and medical history, were obtained by reviewing participants' medical records. Fasting blood samples were obtained for the analysis of lipid profile, glycated hemoglobin A1c (HbA1c), and glucose levels using standard methods at the Department of Clinical Laboratory at Tongji Hospital.

Electrocardiographic recordings were acquired using 24-hour electrocardiogram (ECG) Holter monitoring (DMS 300-4, Holter Reader, Producer DMS, Nevada, USA) in all patients. Parameters from Holter recordings were automatically analyzed by a computer software, which computed all of the basic HRV parameters using the HRV analysis module. The HRV time-domain variables used were as follows: SDNN expressed in milliseconds (ms) represented the standard deviation of all normal-to-normal intervals. SDANN expressed in ms represented the standard deviation of the averages of normal-to-normal intervals in all 5 min segments of the entire recording, and pNN50 represented the ratio of the number of pairs of adjacent normal-to-normal intervals more than 50 ms to the total number of all normal-to-normal intervals.

A standard two-dimensional Doppler echocardiographic examination was performed in all patients according to routine procedures (GE Vingmed Vivid 7 or Vivid 9, Horten, Norway). The following parameters were obtained as previously reported [13]. The left ventricular end-diastolic dimension (LVEDD) was measured using M-mode in the parasternal left ventricular long-axis view. The left ventricular biplane Simpson method was used to measure the ejection fraction (EF) in apical 4- and 2-chamber views. Doppler echocardiography was used to record left ventricular flow velocity and measure LV diastolic function parameters. The peak velocities of early (E-wave velocity) and late (A-wave velocity) transmitral flow were measured, and the E/A ratio was calculated.

### 2.3. Statistical Analyses

Descriptive and experimental measures were expressed as means ± standard deviation or percentages as indicated. The one-sample Kolmogorov-Smirnov test was used to test whether a sample comes from a population with a specific distribution. The Gensini scores showed a markedly skewed distribution toward high values and therefore were presented as medians and quartiles. Correlations between variables were determined using Spearman's correlation or Pearson's correlation coefficient analysis. The analysis of variance test was used for intergroup comparisons of continuous variables. Trends were analyzed with the linear-by-linear association. Two-sided *P* values were used for all tests, and a *P* value < 0.05 was applied to identify the results with statistical significance. All statistical analyses were performed using SPSS version 15.0 for Windows.

## 3. Results

### 3.1. Baseline Characteristics of the Patients

Table 1 summarizes the baseline characteristics, clinical, and biochemical findings of the study population. We included 104 patients with type 2 diabetes and CAD, including 33 females (31.7%) and 71 males (68.3%), with a mean age of 60.6 ± 8.8 years (range 42–77 years). The proportion of patients that reported smoking and alcohol consumption was 47.1% and 32.7%, respectively. The mean body mass index (BMI) was 24.0 ± 2.8 kg/m^2^. Fifty-six patients (53.8%) had hypertension. The mean glycated hemoglobin A1c (HbA1c) value was 6.8 ± 0.6%, and the median Gensini score was 28 (19, 46). The time-domain measures of HRV parameters were as follows: the means of SDNN, SDANN, and pNN50 were 58 ± 19 ms, 101 ± 32 ms, and 11 ± 13%, respectively.

### 3.2. Correlation Analysis according to HRV Parameters

We investigated the association between the HRV parameters and clinical characteristics in all 104 patients. As shown in Table 2, there was no statistical linear correlation between the HRV parameters (SDNN, SDANN, and pNN50) and clinical characteristics (all *P* values > 0.05). Furthermore, we analyzed the correlations between myocardium parameters (E/A ratio, EF, and LVEDD) and HRV parameters (SDNN, SDANN, and pNN50). Results of the linear correlation analysis are also shown in Table 2. However, there were no statistically significant correlations (all *P* values > 0.05).

We further analyzed the possible correlation between the HRV parameters and the severity of coronary lesions as measured both by Gensini scores and the number of affected vessels. As presented in Table 3, there was a significant negative correlation between SDANN and Gensini scores (*r* = −0.22, *P* = 0.03). Notably, after adjustment for clinical covariates, the relationship remained significant (*r* = −0.23, *P* = 0.03). In contrast, no significant correlation was found between the other HRV parameters and Gensini scores (Table 3). Results of the linear correlation between 1-, 2-, and 3-vessel disease and HRV parameters are shown in Table 4. Similarly, there was no statistical correlation between the number of affected vessels and the HRV parameters (all *P* values > 0.05).

## 4. Discussion

In this study, we evaluated the relationship between CAN and the severity of coronary lesions among patients with type 2 diabetes and CAD. CAN is known to be associated with increased cardiovascular morbidity and mortality [22]. The present retrospective study extends these observations by demonstrating that some parameters of HRV were independently associated with the severity of coronary lesions in patients with type 2 diabetes and CAD. These findings suggest that CAN contribute to the development of coronary artery atherosclerosis in patients with type 2 diabetes.

The mechanisms whereby CAN is implicated in the pathogenesis of vascular atherosclerosis are complicated and have not been clarified. Clinical and experimental evidence showed that long-term treatment with antisympathetic drugs resulted in significant reductions of coronary and cerebrovascular complications, thereby reducing mortality and morbidity by the retardation of atherosclerosis development [23]. During the past decade, cardiac autonomic dysfunction has been proposed as a precursor of vascular atherosclerosis development [24]. Autonomic nervous system alterations have been considered as a significant risk factor in the acceleration of atherosclerosis and serve as a trigger for acute coronary and cerebrovascular events. The pathophysiology of CAN is complex and involves the endocrine system with associated inflammatory, hemostatic, and metabolic abnormalities [25]. Although the body of evidence examining the CAN-atherosclerosis association is growing, this association is not fully understood.

It is well known that the diabetes mellitus burden has emerged as an important public health problem of epidemic proportions in the last few decades. In particular, type 2 diabetes mellitus has exponentially grown in the past decades around the world. Clinical studies have demonstrated that type 2 diabetes mellitus can be considered as a CAD risk equivalent with such a strong association and relation with diabetes and CAD development in both men and women [26]. The pathophysiology of type 2 diabetes mellitus in CAD has been attributed to a variety of mechanisms including vascular endothelial injury, atherosclerosis, and CAN. CAN is one of the most common and often overlooked diabetes-related complications that have a significant influence on CAD in patients with type 2 diabetes mellitus [22]. Improving the understanding of the pathogenesis of CAN and its effect on CAD, therefore, has attracted a great deal of interest as a potential therapeutic target.

However, the independent relationship between cardiac autonomic neuropathy and the severity of coronary lesions has been difficult to clarify due to the similar etiologies of these conditions in type 2 diabetes mellitus. Accordingly, we performed the current retrospective study to investigate this important issue. This study showed that the global association of CAN and coronary lesions might be independent of known factors in the pathogenesis of vascular endothelial injury and atherosclerosis in patients with type 2 diabetes mellitus. These findings suggest endothelial dysfunction and cardiac autonomic nervous dysfunction are related pathophysiologically in type 2 diabetes mellitus [27]. Additionally, we indicated that CAN may reflect the progression of coronary atherosclerosis in patients with type 2 diabetes. Previous studies have consistently reported that reduced HRV indexes depend on the degree of damage on coronary artery lesions in patients with stable angina pectoris [28, 29]. Thus, these findings indicate that CAN may reflect the progression of coronary lesions in patients with type 2 diabetes.

A previous study showed that CAN have an independent negative association with exercise capacity in patients with CAD [30]. Moreover, other studies revealed that CAN was associated with left ventricular dysfunction in patients with type 2 diabetes [31–33]. However, we found no significant correlation between HRV indexes and the echocardiographic assessment of LV function in the current study. The lack of association between HRV indexes and echocardiographic assessment of left ventricular function in the present study is not totally surprising, when we consider the complex pathogenesis of these diseases. The divergent findings may be attributed to the heterogeneity of left ventricular dysfunction in patients with type 2 diabetes and CAD. Our study had a few limitations due to the small sample size and the retrospective nature of the study. Moreover, the study was conducted in a single center; therefore, the results cannot be generalized at this point. Notably, future prospective, multicenter, and large-scale studies are warranted to clarify and consolidate our study findings.

In conclusion, we found an association between CAN and the severity of coronary stenosis among patients with type 2 diabetes mellitus and CAD. The combined assessment of cardiac autonomic nervous function and plaque enhancement may improve CAD risk stratification in patients with type 2 diabetes mellitus. Future prospective studies on a larger scale are needed to confirm our findings.

## Figures and Tables

**Table 1:** Demographic characteristics of CAD patients with type 2 diabetes.

Characteristics	Study subjects (*n* = 104)
Men, *n* (%)	71 (68.3)
Age (years)	60.6 ± 8.8
BMI (kg/m^2^)	24.0 ± 2.8
Current smokers, *n* (%)	49 (47.1)
Alcohol drinkers, *n* (%)	34 (32.7)
Hypertension, *n* (%)	56 (53.8)
HbA1c (%)	6.8 ± 0.6
FBG (mmol/L)	7.1 ± 1.2
UA (*μ*mol/L)	416 ± 95
TG (mmol/L)	1.9 ± 1.2
TC (mmol/L)	5.0 ± 0.9
LDL (mmol/L)	3.0 ± 0.9
HDL (mmol/L)	1.1 ± 0.3
EF (%)	57 ± 6
E/A ratio	1.1 ± 0.2
LVEDD (mm)	47.9 ± 4.0
SDANN	101 ± 32
SDNN	58 ± 19
pNN50	11 ± 13
Gensini scores	28 (19, 46)

**Table 2:** The correlations between the HRV parameters and clinical variables.

Variables	SDANN		SDNN		pNN50	
	*r*	*P*	*r*	*P*	*r*	*P*
Age	-0.11	0.25	-0.08	0.41	-0.04	0.71
BMI	-0.03	0.78	0.01	0.98	-0.14	0.16
HbA1c	0.02	0.85	0.01	0.96	-0.01	0.91
FBG	-0.04	0.71	-0.04	0.72	0.12	0.20
UA	0.01	0.98	0.04	0.66	-0.04	0.72
TG	0.04	0.69	-0.08	0.39	-0.01	0.97
TC	0.07	0.51	0.07	0.50	0.09	0.36
LDL	0.08	0.45	0.09	0.35	-0.06	0.54
HDL	0.01	0.90	0.02	0.86	-0.01	0.97
EF (%)	0.07	0.47	-0.01	0.90	-0.14	0.17
E/A ratio	0.13	0.20	0.18	0.07	0.07	0.46
LVEDD	-0.05	0.60	0.17	0.09	0.18	0.06

**Table 3:** Assessment of association between HRV parameters with Gensini scores.

Variables	Crude		Adjust	
	*r*	*P*	*r*	*P*
SDANN	-0.22	0.03	-0.23	0.03
SDNN	-0.14	0.16	-0.10	0.34
pNN50	-0.04	0.67	-0.06	0.60

Spearman's correlation analysis. Adjusted: age, sex, body mass index, smoking, alcohol, hypertension, triglyceride, total cholesterol, HDL, LDL, uric, myocardial parameters, FBG, and HbA1c.

**Table 4:** Correlation between HRV parameters and number of stenotic branches of coronary arteries.

Variables	1VD (*n* = 19)	2VD (*n* = 33)	3VD (*n* = 52)	*P*/*P* for trend
SDANN	101 ± 26	102 ± 26	101 ± 37	0.98/0.97
SDNN	56 ± 18	58 ± 17	58 ± 21	0.91/0.69
pNN50	8 ± 8	8 ± 11	13 ± 16	0.10/0.06

1VD=1-vessel disease; 2VD=2-vessel disease; 3VD=3-vessel disease.

## Data Availability

The data used to support the findings of this study are available from the corresponding author upon request.
